# Unraveling the Complexity of Metabolic Disorders Through Biomarkers: A Focus on Obesity and Type 2 Diabetes Mellitus

**DOI:** 10.3390/biomedicines13102443

**Published:** 2025-10-07

**Authors:** Anna Różańska-Walędziak, Maciej Walędziak

**Affiliations:** 1 Departament of Human Physiology and Pathophysiology, Faculty of Medicine, Collegium Medicum, Cardinal Stefan Wyszynski University in Warsaw, 01-938 Warsaw, Poland; aniaroza@tlen.pl; 2 Department of General, Oncological, Metabolic and Thoracic Surgery, Military Institute of Medicine—National Research Institute, Szaserów 128 St., 04-141 Warsaw, Poland

## 1. Introduction

In recent decades, metabolic disorders, obesity, and type 2 diabetes mellitus have become global health problems. The prevalence of obesity increases every year, and it is associated with a higher risk of developing co-morbidities, including cardiovascular disease, metabolic dysfunction, and type 2 diabetes mellitus, therefore becoming equivalent to metabolic syndrome. Adipose tissue cells release various mediators such as adipokines, cytokines, and chemokines, leading to chronic inflammatory process. Hyperglycemia, insulin tissue resistance, or abnormal lipid profiles are only some examples of metabolic changes in patients with obesity. In recent years, the escalating rates of metabolic disorders have provoked significant concern among healthcare providers, researchers, and policymakers alike. As chronic conditions that affect millions globally, these metabolic disorders impact not only individual health but also pose a tremendous burden on health care systems. The search for effective strategies to combat these conditions is more critical than ever, and the discovery and application of biomarkers have emerged as a promising avenue for improving understanding, diagnosis, and treatment of these intricate diseases.

Biomarkers, defined as measurable indicators of biological processes, offer a window into the physiological status of individuals. In the realm of metabolic disorders, biomarkers can serve multiple roles: they can indicate disease presence (diagnostic), provide insight into disease severity (prognostic), and predict responses to therapeutic interventions (predictive). This multifaceted nature of biomarkers is particularly valuable in conditions like obesity and T2DM, where the disease mechanisms are complex and multifactorial. The spectrum of biomarkers includes traditional measures, such as HbA1c and fasting plasma glucose, as well as more innovative candidates like inflammatory markers, adipokines, and metabolic profiles. Currently, indicators such as HbA1c are instrumental in diagnosing and monitoring T2DM. This biomarker reflects average blood glucose levels over the preceding two to three months, allowing healthcare providers to gauge glycemic control efficiently. However, while HbA1c serves as a cornerstone in diabetes management, it has limitations, particularly in certain populations where its accuracy might be affected by various factors, such as hemoglobin variants or renal disease. In addition to HbA1c, other markers such as fasting insulin levels and Homeostasis Model Assessment of Insulin Resistance (HOMA-IR) are commonly used to assess insulin sensitivity and beta-cell function. Nonetheless, these traditional biomarkers often lack specificity and can miss underlying metabolic dysfunctions, highlighting the need for more comprehensive biomarker panels.

A growing body of research has illuminated the potential of emerging biomarkers that could revolutionize our approach to metabolic disorders [1,2]. Adipokines, for instance, are a group of signaling proteins secreted by adipose tissue, playing a crucial role in metabolic regulation, inflammation, and insulin sensitivity. Biomarkers like leptin and adiponectin have been linked to obesity and T2DM development and progression, offering new avenues for targeted treatment options. Moreover, inflammatory markers such as interleukin-6 (IL-6) and tumor necrosis factor-alpha (TNF-α) have emerged as key players in the inflammatory pathways associated with obesity and insulin resistance. Considering the established link between chronic low-grade inflammation and metabolic disorders, these biomarkers stand at the intersection of immunology and metabolism, presenting unique opportunities for therapeutic interventions focusing on inflammation reduction.

The advent of omics technologies—genomics, proteomics, metabolomics—has further expanded the landscape of potential biomarkers [3]. Through these technologies, we can profile metabolic pathways and identify specific metabolites—such as branched-chain amino acids and specific lipid profiles—that might serve as indicators of metabolic health [4]. These insights are essential for moving towards a personalized medicine approach, where treatment strategies are tailored to the biological contours of each individual [5]. The integration of novel biomarkers into clinical practice promises to enhance the diagnosis and management of metabolic disorders in several critical ways. Innovative biomarkers can facilitate early identification of metabolic dysfunction before the onset of overt disease, allowing for preemptive lifestyle and therapeutic strategies [6]. By identifying individuals at high risk for complications such as cardiovascular disease, biomarkers enable healthcare providers to prioritize interventions that could mitigate these risks [7]. Understanding an individual’s unique biomarker profile allows for tailored therapeutic approaches, optimizing treatment effectiveness and minimizing adverse effects. Biomarkers can provide real-time feedback on the effectiveness of interventions, enabling adjustments to treatment plans based on an individual’s response.

Despite the tremendous potential of biomarkers in managing obesity and T2DM, significant challenges remain. The validation of emerging biomarkers across diverse populations is crucial; a biomarker’s effectiveness can vary based on genetic backgrounds, environmental factors, and lifestyle habits. Rigorous clinical trials are needed to establish standardized protocols for biomarker testing and interpretation, ensuring that the resulting data are robust and applicable to real-world settings. Additionally, the healthcare system must evolve to incorporate biomarker testing into routine care. This requires not only investment in research but also education and training for healthcare providers to understand and interpret biomarker data effectively.

## 2. An Overview of Published Articles

The article by Chen et al. [8] aims to identify glycolysis-associated key genes driving MAFLD progression and elucidate their crosstalk with immune infiltration through bioinformatics analysis and experimental validation. The authors identified five glycolysis-related key genes in MAFLD and explored their relationship with immune infiltration, providing new insights for diagnosis and metabolism-directed immunomodulation strategies in MAFLD. Integrative multi-omics analysis was performed on bulk RNA-seq, single-cell RNA-seq, and spatial transcriptomic datasets from MAFLD patients and controls. Differential expression analysis and WGCNA were employed to pinpoint glycolysis-correlated key genes. The relationship with immune infiltration was analyzed using single-cell and spatial transcriptomics technologies. Machine learning was applied to identify feature genes for matching shared TFs and miRNAs. External cohort validation and in vivo experiments (methionine choline-deficient diet murine models) were conducted for biological confirmation. Genes ALDH3A1, CDK1, DEPDC1, HKDC1, and SOX9 were identified and validated as MAFLD discriminators, with the hepatocyte–fibroblast–macrophage axis having constituted the predominant glycolysis-active niche. Spatial transcriptomics showed that CDK1, SOX9, and HKDC1 were colocalized with the monocyte-derived macrophage marker CCR2. Using four machine learning models, four feature genes were identified, along with their common transcription factors YY1 and FOXC1, and the miRNA “hsa-miR-590-3p”. External datasets and experimental validation confirmed that the key genes were upregulated in MAFLD samples [8].

Abubaker et al. [9] analyzed the clinical utility of growth differentiation factor 15 (GDF-15). GDF-15 is a member of the transforming growth factor-β (TGF-β) superfamily, which is upregulated under cellular stress conditions and has emerged as a potential biomarker for metabolic disorders. However, its expression in relation to diabetes and obesity across different demographic groups remains understudied. This study investigated the association between plasma GDF-15 levels, diabetes mellitus, and obesity in individuals of varying ages, ethnicities, and genders. Plasma GDF-15 concentrations were measured in 2083 participants enrolled in the Kuwait Diabetes Epidemiology Program (KDEP). The dataset included anthropometric, clinical, biochemical, and glycemic markers. Mean plasma GDF-15 levels were significantly higher in males than females (580.6 vs. 519.3 ng/L, *p* < 0.001), and in participants >50 years compared to those <50 years (781.4 vs. 563.4 ng/L, *p* < 0.001). Arab participants had higher GDF-15 levels than South and Southeast Asians (597.0 vs. 514.9 and 509.9 ng/L, respectively; *p* < 0.001). Positive correlations were found with BMI, waist and hip circumferences, blood pressure, insulin, and triglycerides; negative correlations were observed with HDL cholesterol. Median regression indicated that elevated GDF-15 levels were independently and significantly associated with male gender, older age, obesity, diabetes, and insulin resistance. Adjusted median regression indicated that male gender (β = 30.1, 95%CI: 11.7, 48.5), older age (β = 9.4, 95%CI: 8.0, 10.7), and insulin resistance (β = 7.73, 95%CI: 1.47, 14.0) indicated a significant positive association with GDF-15. South Asian participants (β = −41.7, 95%CI: −67.2, −16.2) had significantly higher but Southeast Asian participants (β = −23.3, 95%CI: −49.2, 2.56) had marginally significantly lower GDF-15 levels compared to participants of Arab ethnicity. Higher GDF-15 levels were found to be associated with age, male gender, Arab ethnicity, obesity, and diabetic traits. These findings support the potential role of GDF-15 as a biomarker for metabolic disorders, particularly in high-risk demographic subgroups [9].

Mohamed-Mohamed et al. [10] aimed to examine the association between body composition and glucose tolerance in young adults with normal weight, overweight, or obesity. This observational case–control study included 154 healthy individuals aged 18–25 years. Participants were categorized into three BMI-based groups and underwent anthropometric and body composition assessments using bioelectrical impedance. Glucose tolerance was evaluated via oral glucose tolerance testing, with capillary blood samples collected at baseline and at 30, 60, 90, and 120 min post load. Compared to the normal-weight group, overweight and obese individuals exhibited significantly higher body weight, BMI, visceral and total fat percentages, and reduced muscle mass. Obese participants also showed a significantly greater glucose area under the curve (AUC) and higher fasting and post-load glucose levels. Visceral fat was positively correlated with metabolic impairment. These results indicate a progressive decline in glucose tolerance associated with increasing adiposity and reduced lean mass. Young adults with elevated BMI already demonstrate marked alterations in body composition and impaired glucose tolerance, even in the absence of overt metabolic disease. These findings underscore the importance of the early identification of at-risk individuals using simple, non-invasive tools. Preventive strategies promoting healthy body composition in early adulthood may reduce the future risk of diabetes and its associated complications [10].

The research by Sampedro-Lago et al. [11] evaluated the possible association between serum miR-484 levels and fruit intake frequency with the risk of T2DM in the Spanish adult population. Although evidence suggests that miR-484 and several fruit components are involved in glucose metabolism and insulin resistance metabolic pathways, the relationship between serum miR-484 levels and fruit consumption in relation to the risk of type 2 diabetes (T2DM) remains elusive. A total of 2234 subjects from the Di@bet.es cohort study without T2DM at baseline were studied. Socio-demographic, anthropometric, and clinical data were recorded, as well as responses to a questionnaire on habits, including frequency of fruit consumption (daily vs. occasional). T2DM was diagnosed at baseline and after 7.5 years of follow-up. Baseline serum miR-484 levels were measured using real-time qPCR and categorized based on the 25th percentile. Association analyses were performed using logistic regression models adjusted for potential confounders. Interaction effects were evaluated on the multiplicative and additive scales. There was no association found between miR-484 levels and fruit intake frequency. However, categorized miR-484 levels and fruit consumption were inversely and independently associated with the likelihood of incident T2DM. Analysis of the interaction effect suggests the presence of both positive multiplicative and additive interactions between miR-484 categories and fruit consumption frequency. The study demonstrated a protective effect of daily fruit intake and high miR-484 levels regarding the risk of T2DM and supports the nutritional recommendations advocating daily fruit consumption, as well as suggested that the combined effect of low miR-484 levels and occasional fruit intake may increase the risk of T2DM beyond their independent effects [11].

Al-Lahham et al. conducted research on glycated albumin (GA) [12]. GA serves as a biomarker for short-term glycemic control (2–3 weeks), playing a role in diabetes management. The authors aimed to establish reference intervals (RIs) for serum GA, and the ratios of 1,5-anhydroglucitol to GA (AGI) and GA to HbA1c in a Euro–Brazilian pediatric population (10 y, n = 299), adults (43.5 y; n = 290), and pregnant women (26 y, n = 406; 26.5 ± 3.1 gestation weeks). Both non-pregnant and pregnant women exhibited GA RIs of 10.0–13.3% and 10.6–14.7%, respectively. The AGI ratio varied in the range of 1.2–4.3 in children, 0.9–3.6 in adults, and 0.8–3.1 in pregnant women. Meanwhile, the GA/HbA1c ratio ranged from 1.8 to 2.6 in children and adults to 2.3 to 3.6 in pregnant women. GA and AGI ratios accurately differentiated between T1D and T2D, demonstrating high sensitivity (>84%) and specificity (>97%), with AGI showing superior performance (AUC > 0.99). The GA/HbA1c ratio exhibited moderate discriminatory power (AUC > 0.733) but was less effective in distinguishing adult-onset T1D and T2D, suggesting its limited utility in certain groups. The results of the study confirmed the relevance of RIs for Euro–Brazilian patients. The GA and AGI ratios emerge as valuable diagnostic tools for T1D and T2D, though their reduced sensitivity in diagnosing GDM warrants further investigation. Clinicians might leverage GA and AGI ratios for more tailored diabetes management, especially when HbA1c results are not optimal [12].

The research by Karacic et al. [13] aimed to investigate the validity of the Firmicutes-to-Bacteroidetes (F/B) ratio as a potential biomarker of dysbiosis associated with obesity. The phyla Firmicutes and Bacteroidetes are the main constituents of the gut microbiota. An imbalance in the gut microbiota is a sign of dysbiosis, and the Firmicutes-to-Bacteroidetes ratio has been proposed to be a marker of it, especially in the context of obesity. Since Croatia is the country with one of the highest obesity rates in Europe, a pilot observational study was conducted. Gut microbiota composition was analyzed using 16S rRNA sequencing. The F/B ratio was calculated and evaluated in the context of health factors. No association between the F/B ratio and excess body weight (kg) was found. Excess body weight was significantly associated with higher age, male gender, and history of appendectomy. No significant health predictors of the F/B ratio were found, but weight gain was positively associated with a higher average F/B ratio. Although the study could not confirm the predictive value of the F/B ratio or any other phyla-related biomarker for excess body weight in the study population, it demonstrated interesting insights into the obesity-associated gut microbiota [13].

The review article by Dobriceanu et al. [14] examined the role of pentraxin-3 (PTX3) in DM and assessed the impact of pharmacological interventions on its expression. Diabetes mellitus (DM) is a multifactorial metabolic disorder associated with systemic inflammation and vascular complications. PTX3 has emerged as a key biomarker of inflammation and endothelial dysfunction in DM. The review included studies analyzing PTX3 modulation by antidiabetic therapies, such as sodium-glucose cotransporter-2 inhibitors (SGLT-2i), glucagon-like peptide-1 agonists (GLP-1a), and dipeptidyl peptidase-4 inhibitors (DPP-4i), as well as the effects of lifestyle interventions. Clinical and experimental studies demonstrated a strong correlation between PTX3 levels and DM progression. Elevated PTX3 levels were associated with diabetic complications, including nephropathy, retinopathy, and cardiovascular diseases. Antidiabetic drugs showed differential effects on PTX3 expression, with GLP-1a and DPP-4i significantly reducing PTX3 levels, while SGLT-2i displayed a paradoxical increase. Lifestyle interventions, including dietary modifications and weight loss, yielded inconsistent effects, suggesting genetic and metabolic factors influence PTX3 regulation. While pharmacological therapies, particularly GLP-1a and DPP-4i, demonstrate anti-inflammatory effects, further research is needed to standardize PTX3 measurement and explore its potential as a therapeutic target. Personalized treatment strategies incorporating genetic profiling may optimize inflammation control and disease management in DM patients [14].

Biegański et al. [15] reviewed the role of omentin in obesity, metabolic syndrome, and other diseases. Omentin (omentin-1, intelectin-1, ITLN-1) is an adipokine considered to be a novel substance. Many chronic, inflammatory, or civilization diseases are linked to obesity, in which omentin plays a significant role. MEDLINE and SCOPUS databases were searched using the keywords “omentin” or “intelectin-1”. Then the most recent articles providing new perspectives on the matter and the most important studies, which revealed crucial insight, were selected to summarize the current knowledge on the role of omentin in a literature review. The valid role of this adipokine is evident in the course of metabolic syndrome. In most cases, elevated omentin expression is correlated with a better prognosis in the course of diseases including type 2 diabetes mellitus, polycystic ovary syndrome, rheumatoid arthritis, metabolic dysfunction-associated steatotic liver disease, Crohn’s disease, ulcerative colitis, atherosclerosis, and ischemic stroke, for some of which it can be a better marker than those currently used. However, results of omentin studies are not completely one-sided. It was proven to participate in the development of asthma and atopic dermatitis and to have different concentration dynamics in various types of tumors. All of omentin’s effects and properties make it an attractive subject of research, considering still unexplored inflammation mechanisms, in which it may play an important role. Omentin was proven to prevent osteoarthritis, hepatocirrhosis, and atherosclerosis in mouse models. All of the above places omentin among potential therapeutic products, and not only as a biomarker. However, the main problem with the current state of omentin research is the lack of standardization, which causes many contradictions and disagreements in this field [15].

The article by Mukherjee et al. [16] explored essential biomarkers linked to metabolic dysfunction-associated steatohepatitis (MASH) and type 2 diabetes mellitus (T2DM) to reveal their potential for advancing disease treatment and address their notable overlap. The connection between MASH, obesity, and T2DM highlights the need for an integrative management approach addressing mechanisms like insulin resistance and chronic inflammation. Obesity contributes significantly to the development of MASH through lipid dysregulation, insulin resistance, and chronic inflammation. Selective biomarker targeting offers a valuable strategy for detecting these comorbidities. Biomarkers such as CRP, IL-6, and TNF-α serve as indicators of inflammation, while HOMA-IR, fasting insulin, and HbA1c are essential for evaluating insulin resistance. Additionally, triglycerides, LDL, and HDL are crucial for comprehending lipid dysregulation. Despite the growing importance of digital biomarkers, challenges in research methodologies and sample variability persist, necessitating further studies to validate diagnostic tools and improve health interventions. Future opportunities include developing non-invasive biomarker panels, using multiomics, and using machine learning to enhance prognoses for diagnostic accuracy and therapeutic outcomes [16].

Rausch et al. [17] analyzed the effects of adipose tissue dysregulation on type 2 diabetes mellitus. Internationally, the prevalence of type 2 diabetes mellitus (T2DM) and obesity rates are increasing significantly. As these epidemics continue to spread, the continuation of further research is paramount given that chronic diseases, such as T2DM, cause strain on both economies and healthcare systems. Recently, adipose tissue has been identified as an endocrine organ that produces many hormones that influence many bodily processes. Adipose tissue dysregulation (ATD)—when adipokines (adipose tissue hormones) are produced in abnormal amounts—plays an important role in T2DM development, progression, and prognosis. This narrative review focuses on mechanisms linking ATD with T2DM through adipokine actions (specifically, leptin, and adiponectin) on insulin resistance and glucose metabolism. Here we show that the adipokines leptin and adiponectin are valuable in monitoring, diagnosing, and treating diseases. Further, the leptin-to-adiponectin ratio (LAR) may be more valuable than either adipokine individually. The LAR may give researchers the ability to utilize a primary prevention approach by utilizing LAR as a biomarker influencing early prognosis and treatment. Targeting ATD through diet, weight loss, physical activity, etc., may improve prevention and management outcomes for patients living with or at risk of T2DM [17].

## 3. Discussion

Metabolic disorders such as obesity and type 2 diabetes mellitus pose significant health challenges globally, necessitating a deeper understanding of their underlying mechanisms and potential biomarkers for effective intervention. Recent studies have highlighted the intricate interplay between metabolic pathways, genetic predispositions, and environmental factors that contribute to these conditions. For instance, Kirichenko et al. explored the role of adipokines in obesity-induced inflammation and their potential as biomarkers for early detection of metabolic disorders [18]. Similarly, a comprehensive review by Kahn et al. emphasized the necessity of identifying predictive biomarkers for T2DM risk, advocating for multi-omics approaches to unravel complex biological interactions [19]. More recent work by Ejtahed et al. has demonstrated the potential of gut microbiota profiles in obesity, suggesting that specific microbial metabolites may serve as biomarkers for metabolic health [20]. Additionally, studies like those by Bjornstad et al. have illustrated how imbalances in lipid metabolism can lead to insulin resistance, highlighting possible lipidomic markers for monitoring disease progression [21]. The significance of inflammatory markers has also been underscored by research such as that of Lee et al. who illustrated how elevated levels of C-reactive protein correlate with obesity and are indicative of worsening metabolic health [22]. Furthermore, genetic studies have identified single nucleotide polymorphisms (SNPs) associated with T2DM risk, detailed by Xue et al. paving the way for genetic biomarkers in assessing individual risk profiles [23]. The utility of circulating biomarkers like proinsulin, described by Kosmas et al., has been investigated for their predictive value in insulin resistance and T2DM [24]. In a contrasting approach, research by Briançon-Marjollet et al. has focused on the role of sleep quality and its biomarkers in metabolic regulation, revealing intricate connections between circadian rhythms and metabolic pathways [25]. Collectively, these studies represent a wealth of data reinforcing the notion that a robust framework of biomarkers is essential for unraveling the complexities of metabolic disorders. As clinical practices evolve towards personalized medicine, continuous efforts to validate and integrate these biomarkers will be crucial in targeted prevention and management strategies for obesity and T2DM, as highlighted by Ortiz-Martinez et al. and their exploration of biomarker-guided interventions [26]. As such, interdisciplinary research combining genomics, proteomics, and metabolic profiling will undoubtedly play a key role in enhancing our understanding of metabolic disorders [27,28,29]. These findings emphasize the multidimensional nature of obesity and T2DM and the indispensable role of biomarkers in navigating their complexity for better health outcomes [4,30].

## 4. Conclusions

As we confront the global epidemic of obesity and type 2 diabetes mellitus, biomarkers emerge as beacons of hope. They hold the potential to transform our approach to these complex metabolic disorders, guiding us toward early intervention, personalized treatment, and improved patient outcomes. The integration of traditional and emerging biomarkers into clinical practice reflects a significant leap toward precision medicine in the realm of metabolic health.

Moving forward, it is imperative that we foster collaborative efforts among researchers, clinicians, and policymakers to harness the full potential of biomarkers. This collaboration should focus on validating new biomarkers and developing comprehensive strategies for their incorporation into clinical settings. Public health initiatives must also be strengthened to emphasize the importance of preventive measures that can stop the rising tide of obesity and T2DM—an endeavor that combines the power of biomarker research with community engagement and education.

In conclusion, the journey toward better management of metabolic disorders is multifaceted and requires an unwavering commitment to research, innovation, and collaboration. Biomarkers are not merely tools; they are integral pieces of a larger puzzle that can illuminate the path toward understanding and conquering obesity and type 2 diabetes. By embracing these advancements, we can transform the landscape of metabolic health, ensuring that individuals are equipped with the knowledge and resources necessary to lead healthier, more fulfilling lives. The time to act is now, as the convergence of science and clinical practice paves the way for a brighter future in metabolic disorder management.
